# Cortisol Stability at 16 and 30 Years in Urine Specimens Stored at −20°C

**DOI:** 10.1002/ajhb.70249

**Published:** 2026-04-02

**Authors:** Anwesha Pan, Madison Saunders, Darryl J. Holman

**Affiliations:** ^1^ Department of Anthropology University of Washington Seattle Washington USA; ^2^ Center for Studies in Demography and Ecology University of Washington Seattle Washington USA; ^3^ Utah State University Logan Utah USA; ^4^ Center for Statistics and the Social Sciences University of Washington Seattle Washington USA

**Keywords:** Bangladesh, cortisol, specific gravity, stress, urine

## Abstract

**Objective:**

This study aims to investigate whether urinary cortisol can be reliably measured after long‐term storage at −20°C, using urine samples collected in 1993 from rural Bangladeshi women, and assayed after 16 and 30 years (2009 and 2023).

**Methods:**

Specimens were collected by participants, placed in coolers with ice packs, transported to a field lab, and then refrigerated for up to 1 week before processing. Specific gravity was measured, and a 6.5 mL sample was archived at −20°C. Urinary cortisol was assayed in 2009 as part of a prior study using enzyme‐linked immunosorbent assay (ELISA). In 2023, cortisol was measured in a subsample of 200 specimens using the same assay.

**Results:**

Of the 200 specimens, 7 (3.5%) were measured with low precision. Cortisol could not be detected in nine specimens (4.5%). In the remaining 184 specimens, urinary log cortisol levels measured in 2009 and 2023 were positively correlated (*r* = 0.973, 95% CI: 0.964–0.980; *p* < 0.0001 and nonparametric *r*_s_ = 0.795; *p* < 0.0001). A paired *t*‐test revealed a bias toward slightly greater values in the 2023 measurements (*p* < 0.0001).

**Conclusion:**

These results suggest that urinary cortisol remains largely intact and measurable in specimens stored at −20°C for three decades, and there is a strong correlation between cortisol measured after 16 and 30 years in storage.

## 1 Introduction

Cortisol is a steroid hormone produced from the zona fasciculata of the adrenal gland (Knezevic et al. 2023). The synthesis of cortisol is controlled by the hypothalamus–pituitary–adrenal (HPA) axis (Sheng et al. 2021). Thus, cortisol secretion is directly and indirectly associated with the production of a pituitary‐secreted adrenocorticotropic hormone (ACTH) and hypothalamus‐secreted corticotropin‐releasing hormone (CRH) (El‐Farhan et al. 2017). When the human body perceives stress, it releases CRH, which further triggers the pituitary gland to secrete ACTH (Knezevic et al. 2023). Secretion of ACTH triggers the adrenal cortex to secrete cortisol (Kageyama et al. 2021).

The secretion of cortisol from the adrenal gland follows a circadian pattern (Mohd Azmi et al. 2021). The level of cortisol peaks in the morning and reaches a nadir in the evening (Ramamoorthy and Cidlowski 2016). Cortisol is released in an ultradian, pulsatile pattern (Mohd Azmi et al. 2021). Under stress exposure, the HPA pulsatile system becomes dysregulated and can affect cellular function (Karin et al. 2020). Previous research suggests that exposure to psychological, immunological, and physical energy‐related stressors and exposures results in higher circulating cortisol in the human body (Segerstrom and Miller 2004; Yaribeygi et al. 2017; Dickerson and Kemeny 2004; James et al. 2023). Given that cortisol has a vital role in the pathophysiology of psychological stress, it is a well‐known biomarker of psychological and physical stress in clinical and demographic research as well as investigations of human biology and ecology (Dziurkowska and Wesolowski 2021; Piazza et al. 2010; Hellhammer et al. 2009; Lawes et al. 2022; Bae et al. 2019; Matyas et al. 2024).

Cortisol can be measured in serum, plasma, saliva, hair, and urine samples (El‐Farhan et al. 2017; Iqbal et al. 2023; Turpeinen and Hämäläinen 2013). Urine sample collection is relatively noninvasive, so it can be easier to collect urine samples than blood samples for some purposes (Kapoor et al. 2012). Collection of urine samples (like saliva sampling) minimizes participants' risk of infection and eliminates the risk of pathogenic infections to researchers from sharps used to collect blood samples (Miller et al. 2004). That said, the collection of all three types of fluid specimens entails some risk to researchers through fluid contact with nonintact skin or the mucosa of the eyes, nose, or mouth (Mengistu et al. 2022). Participants can self‐collect and store urine samples, making the method suitable for studies requiring larger sample sizes or serially collected samples (Lloyd et al. 2020).

Cortisol measured in urine averages, over time, the acute circulating levels measured in blood or saliva (Short et al. 2016). Cortisol in urine conjugates with glucuronide and sulfates (Karachaliou et al. 2023). As a result, urinary cortisol measurements may reflect not only circulating levels but also metabolic and excretory differences. Individual differences in glucuronide binding may add variability in the measured concentrations and should be considered when interpreting the results.

Urine samples can be stored frozen for long periods before measurement of analytes (Petrucci et al. 2024), obviating the need to analyze samples immediately after collection. The stability of analytes, including steroid hormones in urine and other biological samples, may be affected by factors such as sample collection procedure, the climate where collection took place, the addition of preservatives to minimize bacterial growth, storage duration, storage conditions, storage temperature, the period between collection and lab analysis, and the number of freeze–thaw cycles preceding analysis (Remer et al. 2014; Rotter et al. 2017). For example, cortisol and other hormones stay intact when stored in the freezer for a long duration (Moffat et al. 2020). On the other hand, storage duration may degrade salivary cortisol concentrations over time (Rosenbaum et al. 2018).

Few studies have explored the stability of cortisol in samples stored frozen over decades. Most such studies have investigated stability in saliva and plasma samples (Kley et al. 1985; Garde and Hansen 2005). One study demonstrates the stability of cortisol for 10 years in urine samples stored frozen at −80°C (Moffat et al. 2020).

Here, we examine the stability of cortisol in urine samples stored at −20°C for 30 years and measured at Years 16 and 30. The urine samples were collected during a field study of rural Bangladeshi women in 1993.

## 2 Materials and Methods

### 2.1 Sample Collection

The urine samples were collected in Bangladesh as a part of a field study of fecundity (Holman 1996). The research received IRB approval from the Pennsylvania State University Office for Regulatory Compliance and the International Centre for Diarrhoeal Disease Research, Bangladesh (ICDDR, B). The study was conducted in Matlab Thana, a rural administrative unit (comparable to a US county) located 55 km southeast of Bangladesh's capital, Dhaka, from February to December 1993. Trained field workers interviewed married women aged 18–48 years in 28 villages in Matlab. There was no economic incentive for the study participants. The inclusion criteria of the study were married reproductive‐aged women residing with husbands and not using contraception.

A questionnaire was administered, and urine samples were self‐collected twice weekly from 492 women for at least 1 month and up to 9 months under the guidance of female field workers at the participants' homes. Specimens were collected in Fisher Scientific 120 mL plastic specimen cups. Most samples were collected in the morning. Urine specimens were placed in a portable cooler immediately after collection and moved to a large chest cooler in the field worker's home after her daily round. Ice packs were replaced in coolers every 2–3 days. At the same time, urine specimens were transported to a rural Matlab hospital and refrigerated. The urine samples were brought to room temperature after 1–3 days to measure pH (Horiba C‐1 pH meter, Horiba Ltd., Tokyo, Japan) and SpG (Atago Uricon‐N urine specific gravity refractometer; NSG Precision Cells Inc., Farmingdale, NY, USA). A 6.5 mL sample of each urine specimen was taken and preserved with 17 g/L boric acid solution, transferred into a Fisherbrand 7 mL HDPE Scintillation Vial, and stored at −20°C. These preserved samples were later transported frozen via overnight air freight to the United States. They were stored at (−20°C) at the Pennsylvania State University until 1999, when they were transported frozen to the University of Washington Biodemography lab and stored at (−20°C).

The specimens underwent from two to five freeze–thaw cycles with no more than 2 weeks at refrigerated temperature and no more than 1 day at ambient temperature. Most research suggests that cortisol is relatively resilient to freeze–thaw cycles in urine (Endo et al. 2025; Leoni et al. 2025; Wang et al. 2018), saliva (Garde and Hansen 2005; Sontag et al. 2023), and serum and plasma (Tjernvoll et al. 2025).

### 2.2 Urinary Cortisol Levels Measurement in 2009

In 2009, cortisol was assayed on 4174 specimens collected from 236 women experiencing postpartum amenorrhea in the University of Washington Biodemography Lab. Cortisol concentration was measured by an in‐house ELISA immunoassay (Konishi et al. 2012; O'Connor et al. 2011; Trumble et al. 2010). The assay used a cortisol‐horseradish peroxidase (HRP) ligand and antiserum (the antibody R4866 and conjugate were provided by C.J. Munro, University of California at Davis). Details and validation of the urinary assay can be found elsewhere (McCallister et al. 2004; Skurski 2006). Cross‐reactivity of this antiserum was: cortisol 100%, prednisolone 9.9%, prednisone 6.3%, cortisone 5%, and others,1% with corticosterone, desoxycorticosterone, 21‐desoxycortisone, testosterone, androstenedione, androsterone, and 11‐desoxycortisol (Umapathy et al. 2015). Absorbance was measured with an MR7000 Plate Reader (Dynatech Corp. Burlington, MA, USA) (test wavelength, 405 nm; reference wavelength, 570 nm) (O'Connor et al. 2011). Urine samples were diluted 2‐, 30‐, 60‐, and 120‐fold as needed to measure cortisol within the limits of the assay.

### 2.3 SpG and Cortisol Measurements in 2023

In 2023, SpG and cortisol concentrations were remeasured using the same immunoassay (including the same protocol, batches of antibodies and tracers) in a subsample of 200 urine specimens for which cortisol could be reliably measured. Samples were diluted (EIA block solution) 2‐, 30‐, 60‐, and 120‐fold as needed for the assay. Two differences between the 2009 measurements are that absorbance was measured with a BioTek microplate reader (Synergy HT, Santa Clara, CA, USA) and the SpG of urine specimens was measured in 2023 by a different clinical refractometer (A 300 CL, Atago Co. Ltd., Tokyo, Japan). All urinary cortisol values were corrected by SpG as (SpG_target_ − 1.0)/(SpG_sample_ − 1.0), where the target SpG of the Bangladeshi urine sample is 1.015 (Miller et al. 2004; Holman et al. 2006).

For the paired analysis, we only included samples in which cortisol was reliably measured, as assessed by (1) a result greater than the assay's lower limit of quantification and (2) a coefficient of variation (CV) below 16%. We selected 16% as a conservative upper threshold, consistent with standards in the field where intra‐assay CVs up to 10% are ideal, and values up to 15% may be considered acceptable in large‐scale biomarker studies (Brindle et al. 2021).

### 2.4 Statistical Analysis

Pearson correlations were estimated between SpG measured in 1996 and 2023. Nonparametric Spearman rank correlations and Pearson correlations were estimated for log SpG‐corrected urinary cortisol concentrations measured in 2009 and 2023. A paired *t*‐test of log cortisol concentrations was performed to assess bias. Regression analysis between 2009 and 2023 log SpG‐corrected cortisol concentrations was used to estimate the percent concentration change between the two time points.

## 3 Results

### 3.1 SpG Comparison

**FIGURE 1:**
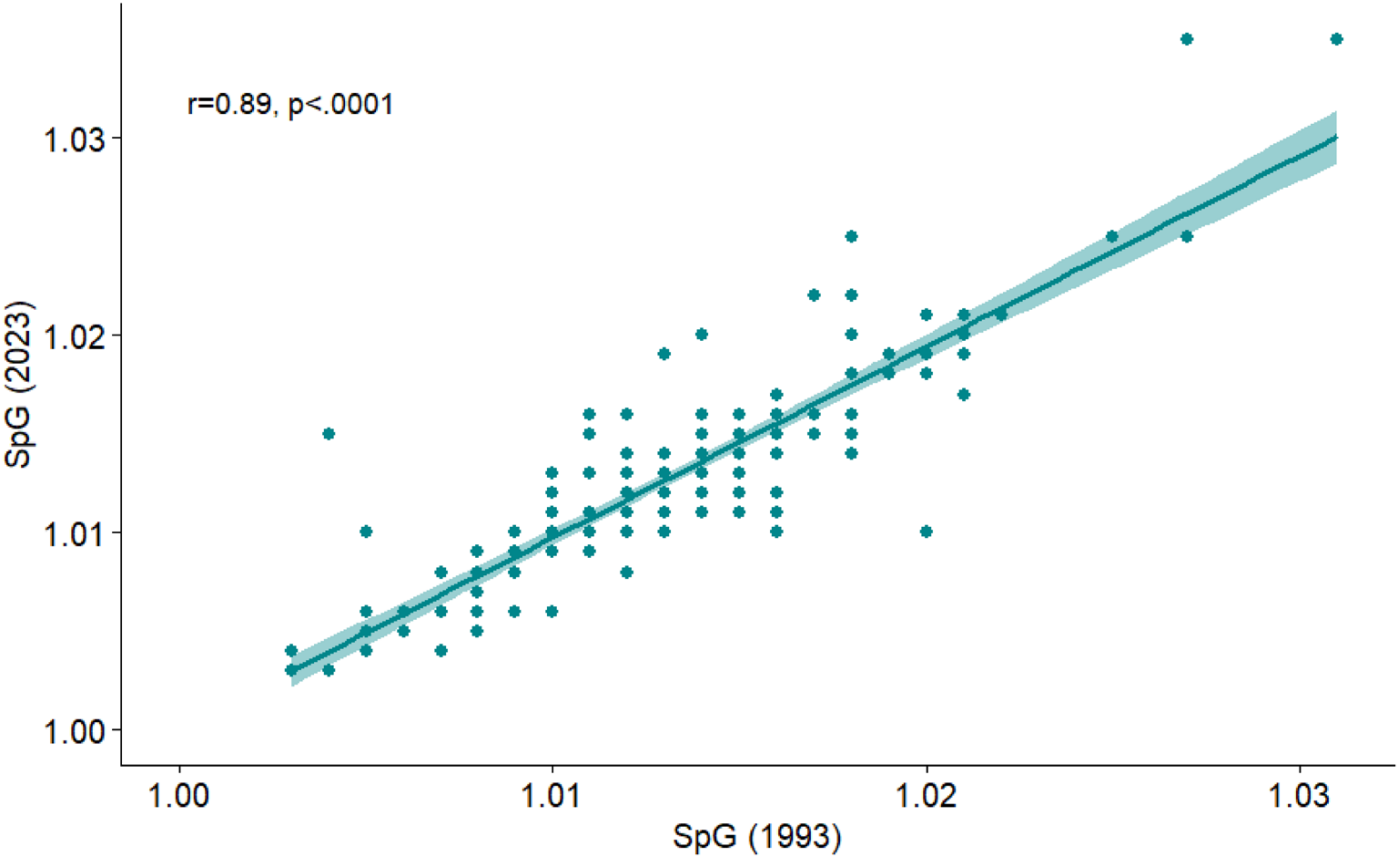
Correlation between SpG measured in 1993 and 2023.

SpG was first measured in 1993, within a week or so of collection, and the values were used to correct the cortisol concentration that had been measured in 2009. SpG was remeasured in 2023. SpG measured in 1993 and 2023 had a strong positive correlation (*r* = 0.892, 95% CI: 0.858–0.918; *p* < 0.0001) (Figure 1). The histograms of SpG measured in 1993 and 2023 are shown in Figure S1. The change in SpG measurement between 1993 and 2023 for each sample is shown in Figure S2. A paired *t*‐test found no significant difference (mean: 0.00034; *p* = 0.056) between SpG measured in 1993 and 2023.

### 3.2 Cortisol Assays

In 2009, cortisol was measurable in 4081 (97.8%) of 4174 specimens assayed. For 93 (2.2%) specimens, cortisol was below the analytical limit of quantification. If we assume 100% of samples had measurable cortisol in 1993, then we can measure *sample attrition* as the percent of samples that no longer have measurable cortisol, and take this as a rough measure of degradation of cortisol over time. We do not know that all samples had measurable cortisol in 1993, but assuming they did gives us a worst‐case estimate for the rate of sample attrition. The inability to measure cortisol in 2.2% of the samples after the first 16 years of storage gives a maximum sample attrition of about 0.14% samples per year.

In 2023, cortisol was reliably remeasured in 184 (92%) of the 200 specimens. Cortisol was below the limit of detection in nine specimens (4.5%), and for seven specimens (3.5%), cortisol was measured but had a CV > 16%. Because all 200 specimens had measurable cortisol in 2009, we take the 4.5% of samples with no measurable cortisol in 2023 to estimate sample attrition over the 14 years of storage from 2009 to 2023 as 0.15% specimens per year, quite similar to the rate we found between 1993 and 2009.

### 3.3 Cortisol Concentrations Comparison

**FIGURE 2:**
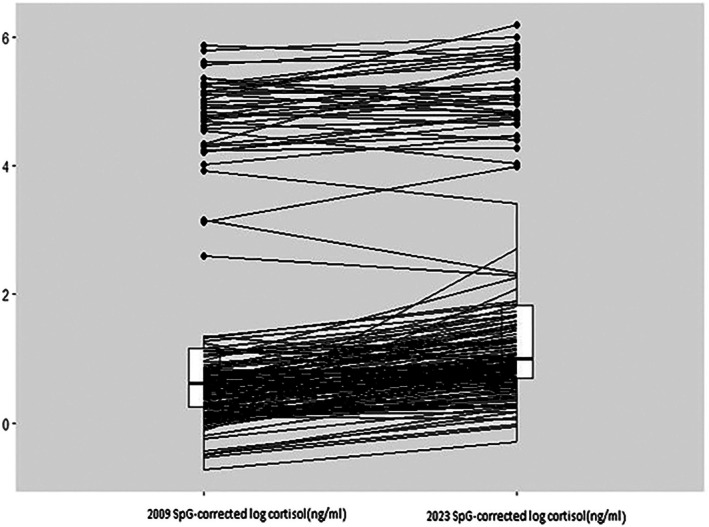
Changes within a sample between 2009 and 2023 in log cortisol concentrations.

**FIGURE 3:**
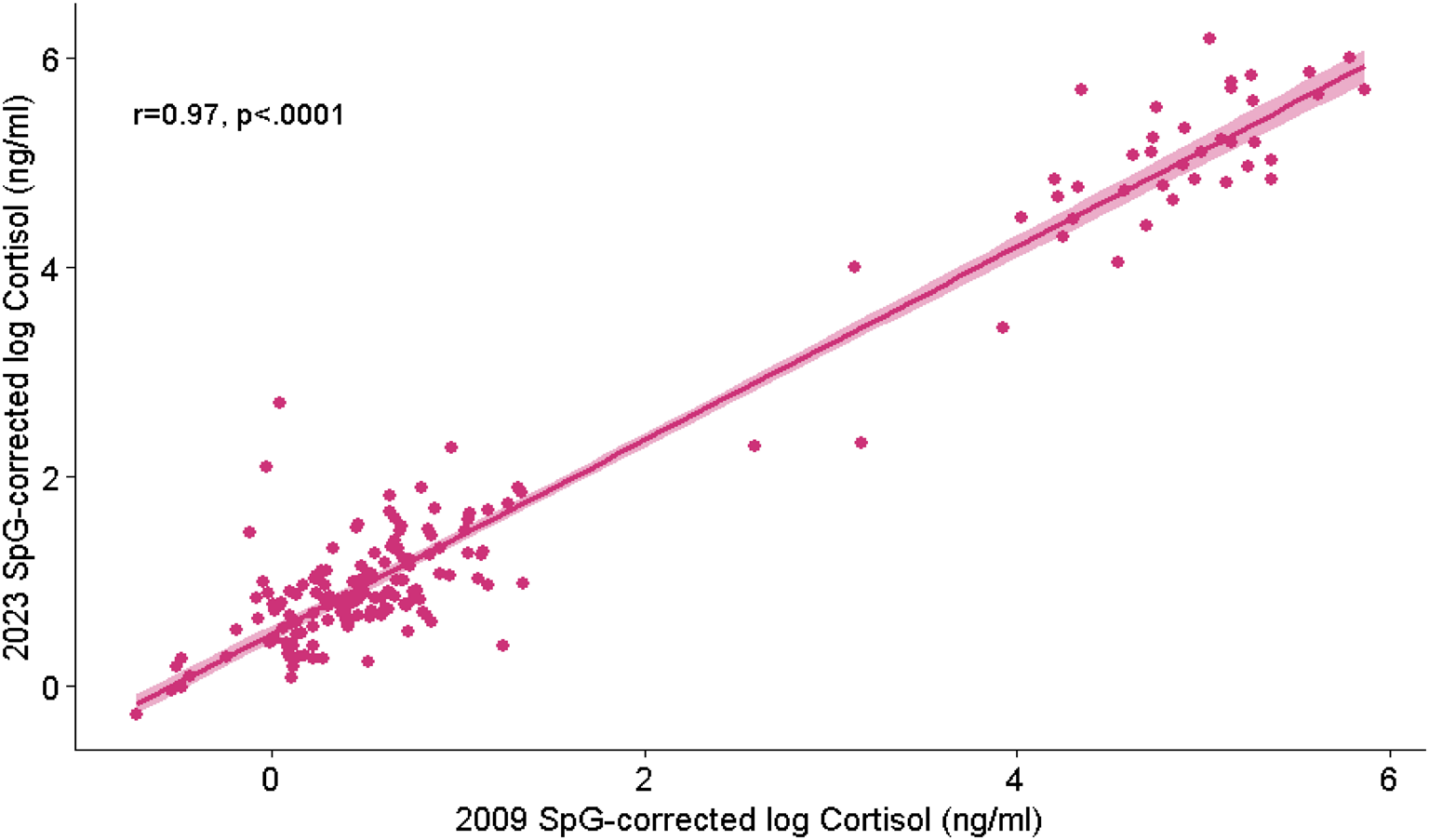
Correlation between SpG‐corrected log cortisol concentration measured in 2009 and 2023.

The change in cortisol measurement between 2009 and 2023 for each sample is shown in Figure 2. Figure 3 suggests a slight increase, on average, in cortisol concentrations in 2023 compared to 2009. This can also be seen in the distributions of measurements shown in Figure S3. SpG‐corrected log cortisol concentration measured in 2009 and 2023 indicated a strong positive correlation (*r* = 0.97, 95% CI: 0.96–0.978; *p* < 0.0001) (Figure 3). The nonparametric correlation between SpG‐corrected log cortisol concentrations measured in 2009 and 2023 was *r*_s_ = 0.795 (*p* < 0.0001).

### 3.4 Bias in Cortisol Concentration Measurements in 2009 and 2023

**FIGURE 4:**
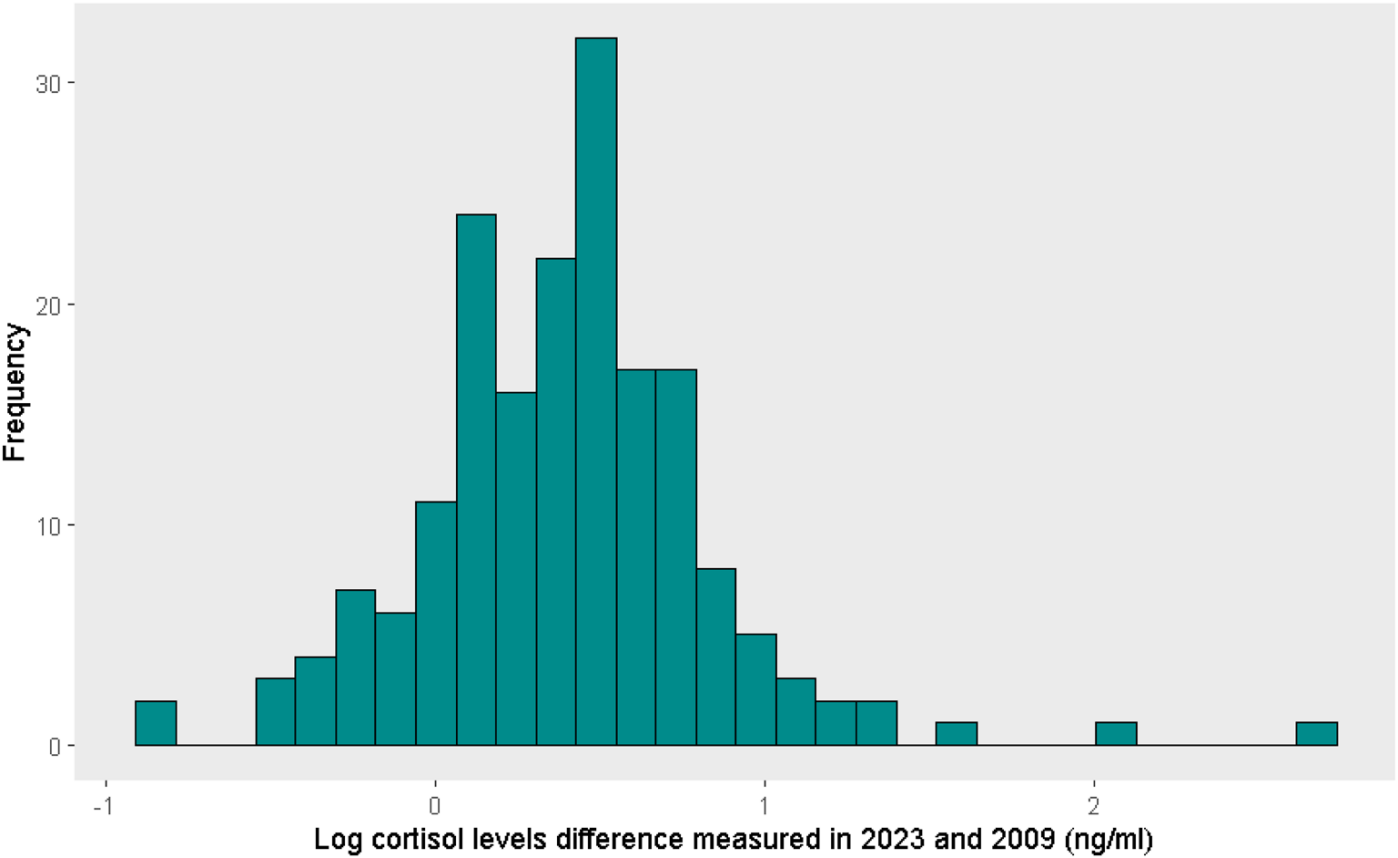
Difference in the SpG‐corrected log cortisol (ng/mL) concentration measured in 2009 and 2023.

A paired *t*‐test found a significant difference between the 2023 and the 2009 SpG‐corrected log cortisol concentration (mean difference: 0.391, 95% CI: 0.326–0.456; *p* < 0.0001) (Figure 4). The geometric mean of the ratio of SpG‐corrected log cortisol concentrations measured in 2023 and 2009 is 2.46 (95% CI: 2.19–2.86). As this geometric mean exceeds 1.0, it indicates greater concentrations of cortisol measured in 2023. These results further support a slight increase in cortisol concentrations in 2023 compared to 2009, as suggested by Figure 2. The linear regression suggests that a 1% increase in 2009 SpG‐corrected log cortisol concentration gives a 1.02% increase in SpG‐corrected log cortisol concentration measured in 2023.

## 4 Discussion

We remeasured SpG in 2023, and we found a high correlation and no significant difference between the 1993 and 2023 measures. This suggests that the specimens did not undergo density changes in 30 years of storage.

Because cortisol was not measured at the time of collection in 1993, this study cannot fully assess cortisol degradation between 1993 and 2009. However, we were able to estimate the maximum sample attrition rate over the first 16 years of storage (0.14% samples per year) and the observed sample attrition rate from 16 to 30 years of storage (0.15% samples per year), based on the number of specimens in which cortisol could not be detected at measurable levels from the first to the second time point. We interpret this as a crude nonparametric measure suggesting a small amount of degradation of cortisol over time under these storage conditions. The strong correlation between measures taken at 16 and 30 years demonstrates that cortisol largely remained measurable and consistent after long‐term storage at −20°C.

We found a small bias toward higher measures of log cortisol concentration between samples measured in 2009 and remeasured in 2023. This bias can potentially result from slight water loss in the urine samples stored frozen for more than 30 years (Beauval et al. 2022; Nauta et al. 2012). On the other hand, any such change in the frozen specimen would likely increase SpG; the net result should be no change in the final measurement. The bias more likely reflects small differences in assay and laboratory procedures (Berger et al. 2024). For example, we used different equipment to measure SpG in 1993 and 2023, and different optical plate readers were used in 2009 and 2023. The bias is small on a log scale and is probably substantively unimportant. All the urine samples were stored under the same conditions for about the same amount of time, and the same lab procedures were followed to the extent possible for hormone measurements. Hence, the bias in measurement of cortisol concentrations should be about the same for all the urine samples (Berger et al. 2024).

By another measure, the 4.5% of 200 samples in which we could not detect cortisol after the 14‐year period of storage suggests some degradation of cortisol in the specimens. Even with both of these differences, a bias toward greater values and possible degradation, these results are encouraging. Our objective was not to demonstrate that concentrations were identical between 2009 and 2023. The high correlation would still demonstrate the usefulness of the specimens even if modest degradation has occurred. In fact, if we had used an assay with a different antibody that cross‐reacted differently to cortisol conjugates, a comparison of measured concentrations would not have been useful, and we would have relied on the correlation to assess whether inferences from 2023 measures would be similar to inferences from 2009 measures. We would expect to lose 4.5% of samples near the lower limits of the assay, but the correlation with the remaining 95.5% should be high.

These results are consistent with previous studies indicating that cortisol can remain stable in frozen biological samples stored over long periods. Research from the mid‐1980s showed the stability of cortisol stored for long periods in biological samples (Kley et al. 1985). That study found that cortisol concentrations declined after 3–4 years of storage but demonstrated the overall stability of steroid hormones in plasma samples stored frozen for more than 10 years (Kley et al. 1985). Other studies using clinical samples also found that cortisol was stable in frozen biological samples stored for long periods. For example, in 2005, Garde and Hansen (2005) demonstrated cortisol stability in saliva samples stored cold. They found about a 9% decrease in cortisol per month in saliva samples at room temperature, but stability for up to 3 months stored at 5°C, and stability for at least a year stored at −20°C or −80°C. Moffat et al. (2020) used 11.9 ± 6.4 years old urine samples stored at −80°C from the Baltimore Longitudinal Study of Aging biobank to assess longitudinal changes in cortisol measurements. Urinary free or unconjugated cortisol was measured by liquid chromatography with mass spectrometry. Their results suggested that cortisol is stable when frozen at −80°C over long periods, even though the study was not designed to evaluate stability. Overall, our results, combined with results from prior studies using different sample matrices and methods, provide compelling evidence that cortisol is stable when stored frozen over long periods. The degree of stability may depend on factors like storage time, matrix type, and temperature.

Few studies have examined the stability of hormones in samples collected in field settings and stored for extended periods. Rosenbaum et al. (2018) explored the effects of storage time and condition on different hormones in saliva samples collected during the Cebu Longitudinal Health and Nutrition Survey. They found that longer storage at −35°C had a weak association with lower values of cortisol (Rosenbaum et al. 2018).

The current study has several strengths. First, this is the only study we are aware of that shows cortisol can be recovered from urine samples stored frozen at −20°C for 30 years. The measurements were highly correlated with measurements taken 14 years earlier. In several Nordic countries and US biobanks, biological samples have been collected from large cohorts and stored at or below −20°C. These results encourage the use of this and other biospecimen archives for future research that uses cortisol. Second, the current study used urine samples collected in a field research setting. With some exceptions (e.g., Rosenbaum et al. 2018), most prior studies examined cortisol stability in samples collected in clinical settings, where conditions are usually more optimal for collection, transportation, preservation, and storage.

The current study has several limitations. Foremost, we did not have cortisol measurements from 1993, when the specimens were collected. Therefore, we must assume that degradation (if any) between 1993 and the first cortisol measurements in 2009 would affect all specimens approximately equally. We also assume that degradation was not severe, relying on two observations. First, the small percentage of samples in which no cortisol could be detected in 2009 and 2023. Second, studies have found that the length of storage of frozen specimens does not cause degradation of cortisol in plasma (Kley et al. 1985), saliva (Garde and Hansen 2005), and urine (Moffat et al. 2020). None of the studies examined urinary specimens stored at −20°C or periods of over 10 years, so they are only suggestive about any degradation in these Bangladesh samples.

We could not assess the effect of past freeze–thaw cycles on cortisol concentrations because no records were kept on freeze–thaw cycles. Each sample was thawed at least two times, but some samples may have had additional cycles. A number of studies have found that freeze–thaw cycles do not substantively affect cortisol concentrations measured from urine (Endo et al. 2025; Leoni et al. 2025; Wang et al. 2018), saliva (Garde and Hansen 2005; Sontag et al. 2023), and serum and plasma (Tjernvoll et al. 2025).

Even with these limitations, we believe that 30‐year‐old archived specimens are still useful for doing some types of research. Clearly, we cannot make inferences about the absolute concentrations of cortisol in 1993. But we believe that any signal found in the cortisol measurements is still relatively intact after 30 years of storage. Thus, studies of the relationship between cortisol and other physiological, environmental, or behavioral traits should yield similar findings that would have been found with fresh samples, assuming the findings do not rely on the small percentage of samples (that likely began with low concentrations of cortisol) that have degraded to below detectable levels. These findings support the utility of archived urine samples for retrospective cortisol measurement research, while highlighting the need for caution when inferring original concentrations.

## Author Contributions

**Anwesha Pan:** conceptualization (equal), data curation (equal), formal analysis (equal), investigation (equal), methodology (equal), software (equal), supervision (equal), validation (equal), visualization (equal), writing – original draft (equal), writing – review and editing (equal). **Madison Saunders:** data curation (equal), investigation (equal), methodology (equal), software (equal), validation (equal), visualization (equal), writing – review and editing (equal). **Darryl J. Holman:** conceptualization (equal), data curation (equal), formal analysis (equal), formal analysis (equal), funding acquisition (equal), funding acquisition (equal), investigation (equal), investigation (equal), methodology (equal), methodology (equal), project administration (equal), project administration (equal), resources (equal), resources (equal), software (equal), software (equal), supervision (equal), supervision (equal), validation (equal), validation (equal), visualization (equal), visualization (equal), writing – review and editing (equal), writing – review and editing (equal).

## Funding

This work was supported by the Center for Studies in Demography and Ecology, University of Washington (P2C HD04282 and T32 HD101442).

## Conflicts of Interest

The authors declare no conflicts of interest.

## Supporting information


**Figure S1:** Distributions of SpG measured in 1993 and 2023.
**Figure S2:** Changes within a sample between 1993 and 2023 in SpG.
**Figure S3:** Distributions of SpG‐corrected log cortisol concentrations measured in 2009 and 2023.

## Data Availability

The authors have nothing to report.
