# Supplementary material for: Cortisol Stability at 16 and 30 Years in Urine Specimens Stored at −20°C
Source: Am J Hum Biol. 2026 Apr 2;38(4):e70249. doi: 10.1002/ajhb.70249 (PMC13044983; doi:10.1002/ajhb.70249)
Supplement: Supplementary file 1 — Figure S1: Distributions of SpG measured in 1993 and 2023. Figure S2: Changes within a sample between 1993 and 2023 in SpG. Figure S3: Distributions of SpG‐corrected log cortisol concentrations measured in 2009 and 2023. [file AJHB-38-e70249-s001.docx]

**Supplementary material**:


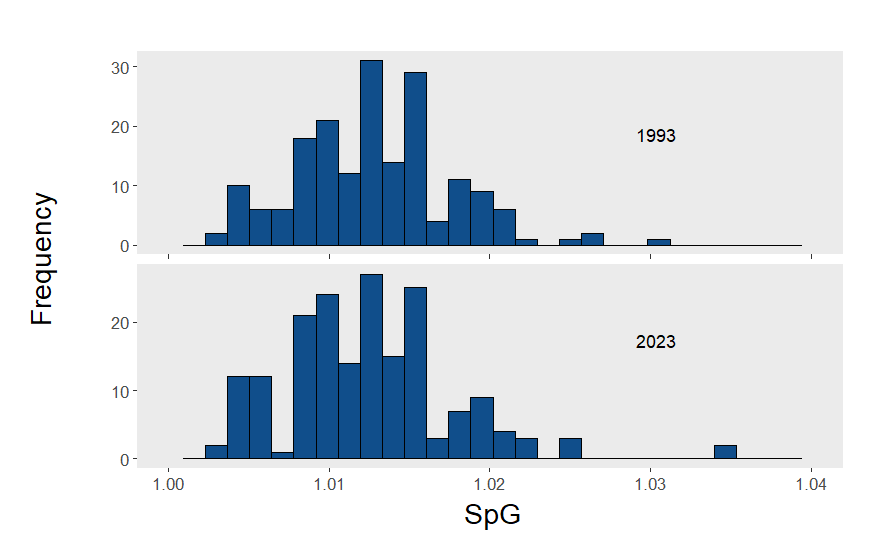


Figure S1. Distributions of SpG measured in 1993 and 2023.


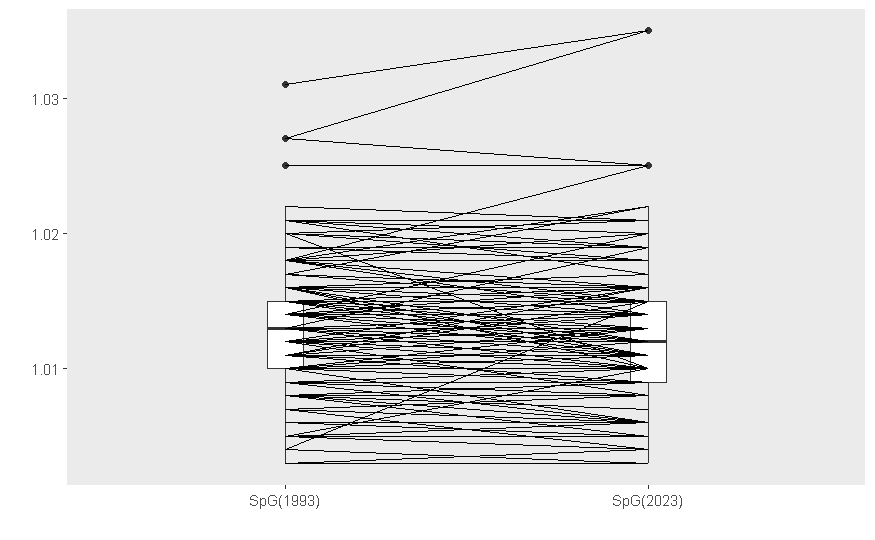
Figure S2. Changes within a sample between 1993 and 2023 in SpG.


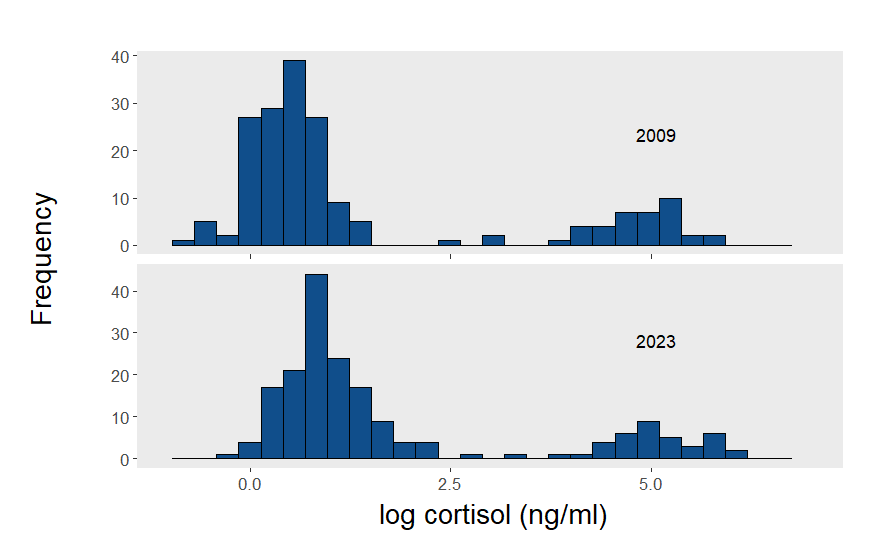


Figure S3: Distributions of SpG-corrected log cortisol concentrations measured in 2009 and 2023.
